# Shotgun metagenomic mapping of saliva reveals insights into diversity and function of the oral microbiome in pregnancy

**DOI:** 10.1038/s41598-026-54100-3

**Published:** 2026-05-27

**Authors:** Nagihan Bostanci, Anusha T. Antony, Angelika Silbereisen, Tina Esmaili, Maria C. Krog, Irene Sterpu, Zahra Bashir, Lars Engstrand, Eva Wiberg-Itzel, Henriette Svarre Nielsen, Luisa W. Hugerth, Ina Schuppe-Koistinen

**Affiliations:** 1 Division of Oral Health and Periodontology, Department of Dental Medicine, Karolinska Institutet, Stockholm, Sweden; 2 Department of Medical Biochemistry and Microbiology, Science for Life Laboratory, Uppsala University, Uppsala, Sweden; 3 The Recurrent Pregnancy Loss Units, Copenhagen University Hospitals, Rigshospitalet and Hvidovre Hospital, Copenhagen, Denmark; 4 Department of Clinical Immunology, Copenhagen University Hospital, Rigshospitalet, Denmark; 5 Department of Clinical Science and Education, and Division of Obstetrics and Gynaecology, Department of Clinical Science, Intervention and Technology, Karolinska Institute, Södersjukhuset, Stockholm, Sweden; 6 Department of Obstetrics and Gynaecology, Holbæk Hospital, Holbæk, Denmark; 7 Department of Microbiology, Tumor and Cell Biology, Centre for Translational Microbiome Research, Karolinska Institutet, Stockholm, Sweden; 8 Department of Obstetrics and Gynaecology, Hvidovre Hospital, Copenhagen, Denmark; 9 Department of Clinical Medicine, University of Copenhagen, Copenhagen, Denmark

**Keywords:** Pregnancy, Oral health, Oral microbiome, Oral ecology, Oral-systemic link, Saliva, Hormones, Shotgun sequencing, Adverse pregnancy outcomes, Vaginal delivery, Cesarean, Microbiology, Molecular medicine, Mathematics and computing

## Abstract

The oral microbiome is a complex and dynamic microecosystem that fluctuates continually throughout the lifespan of a woman. Nevertheless, the function of the oral microbiome in reproductive health is not yet fully understood. Monitoring oral health and providing necessary dental care before and during pregnancy could help maintain a balanced oral microecology and support healthier microbial transfer to newborns. Here, we aimed to compare the salivary microbiome of pregnant and non-pregnant women using shotgun metagenomics to describe their taxonomic and functional composition and assess whether the resulting data is better explained by the reproductive stage. We conducted a comparative cross-sectional study involving pregnant women (n = 71; gestational age 37–42 weeks) and non-pregnant women (n = 143 with regular menstrual cycles; 3 saliva samples per participant across different menstrual phases). Shallow shotgun metagenomic sequencing was used to characterize both taxonomic and functional profiles of the oral microbiome. Socransky’s color complex analysis was performed to assess group differences in key microbial complexes. Quantitative PCR was used to validate the abundance of selected oral bacteria. Participant data, including demographic, behavioral, clinical, and oral health variables (such as dentist visits), were collected and incorporated as covariates to adjust for potential confounding effects. Additionally, a sensitivity analysis was performed by excluding participants with identified behavioral or clinical risk factors. Ten phyla including Actinomycetota, Bacteroidota, Chloroflexota Bacillota, Fusobacteriota, Pseudomonadota, Spirochaetota, Synergistota Candidatus Saccharimonadota and Mycoplasmatota, 102 genera, and 410 species were identified. Pregnant women had lower saliva microbiome diversity, driven by reduced richness but unchanged evenness. The microbial composition varied between the groups, even after adjusting for confounding factors. Differential abundance analysis, adjusted for potential confounders, identified 25 species that significantly differed between groups (q < 0.05), with 13 taxa more than three-fold higher in pregnant women. Notably, red complex species were more abundant in pregnant women (p < 0.05). Functional pathway analysis identified 40 modules that differed by pregnancy status. These results further suggest a connection between pregnancy and changes to the oral microbiome in women. As many of these changes are in a pro-inflammatory direction, further research is warranted to assess its potential impact on pregnant women and their newborns.

**Supplementary Information:**

The online version contains supplementary material available at 10.1038/s41598-026-54100-3.

## Introduction

The pivotal role of microbiota communities in shaping human health has been extensively recognized. While a predominant focus has historically centered on the gastrointestinal and vaginal milieu, there is growing interest in the potential implications of the oral microbiome in the context of female reproductive health and offspring well-being ^1–3^. Nevertheless, there is limited knowledge regarding alterations in the oral microbiome throughout gestation and the potential impact of pregnancy-induced changes on outcomes^4–6^. Oral microbial dysbiosis, coupled with gingival inflammation, has been linked to adverse pregnancy outcomes such as low birth weight, preterm birth, preeclampsia, and pregnancy loss^3,7–10^ . Particularly estrogens have been shown to potentially diminish the physiological salivary flow rate ^11^. This reduction leads to a decline in the innate antimicrobial efficacy of saliva, subsequently disturbing local microbial equilibrium^12,13^. Alterations in the oral microbiome of pregnant individuals are manifest by an elevated prevalence of periodontal and cariogenic pathogens in their oral milieu^14^. These alterations are hypothesized to be driven by pregnancy creating a nutrient environment that is more beneficial to those species ^15^. Concentrations of estradiol and progesterone in saliva are known to increase during pregnancy and decline postpartum^16^. Earlier conventional bacterial culture or quantitative PCR methods have identified overgrowth of selected periodontal pathogens, such as *Porphyromonas gingivalis, Prevotella intermedia, Treponema denticola, Tannerella forsythia, Campylobacter rectus, Prevotella nigrescens*, with pregnancy^17–20^. Additionally, some studies reported that pregnant women experienced a significant increase in *Streptococcus mutans*, a species asscoiatied with onset of dental caries ^21^. Relatively small case–control studies also showed that the levels of oral bacteria species i.e. *Aggregatibacter actinomycetemcomitans, P.gingivalis* tended to increase during pregnancy especially in mothers with preterm birth^22,23^. Although adverse pregnancy outcomes were suggested to induce an overgrowth of selected periodontal pathogens, further larger studies failed to show the quantity of the dental plaque microflora to predict preterm birth ^24^. *Fusobacterium nucleatum* is an oral bacterium that is among several microorganisms associated with preterm birth. The prevalence of *F. nucleatum* correlates with the severity of periodontal diseases, which results in the progression of inflammation. This inflammatory process facilitates the translocation of the organisms to the intrauterine cavity through hematogenous transmission ^25^.

More recent small scale studies evaluated the oral microbiome during pregnancy by 16S rRNA sequencing revealed that the diversity of oral microbiome remains relatively stable during pregnancy; however, pregnancy was associated with pathogenic shift in abundance of specific oral microorganisms when compared to postpartum/non-pregnant status^26,27^.

Nevertheless, a subset of the literature has advanced the notion of negligible disparities in oral microbiome composition between pregnant and non-pregnant individuals ^28^. Consequently, uncertainties remain regarding the precise alterations in the compositional makeup and structural configuration of oral bacteria in the context of pregnancy. Considering the pronounced shifts in emotional, behavioral, and diet changes during pregnancy substantial adaptations are anticipated within the maternal oral microbiota ^29,30^. Nevertheless, most studies on oral microbiome changes in pregnant women have overlooked emotional, behavioral, and diet influences, potentially confounding results. Additionally, the non-pregnant control groups often neglect to account for women’s menstrual cycle status. We have previously reported that the oral microbiome adapts to the endogenous hormonal fluctuations of the menstrual cycle, and this adaptation is influenced by factors such as diet and smoking ^3,31^. While many studies have been carried out on the oral microbial communities of pregnant women, most notably using 16S rRNA gene high-throughput sequencing, its limitations in providing functional insights and species-level resolution have prompted the adoption of shotgun sequencing. Here, we have compared the salivary microbiome of healthy pregnant and non-pregnant women using shallow shotgun metagenomics to describe their taxonomic and functional composition and assess whether the resulting data is better explained by the reproductive stage.

## Materials and methods

### Study design and ethics statement

**Fig. 1:**
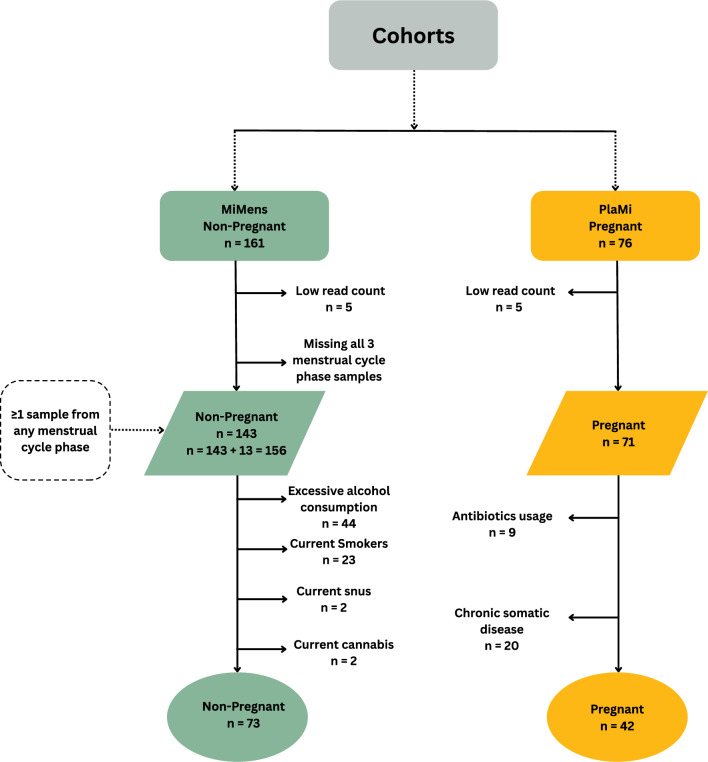
Study design for sample collection and analyses. From the original cohort of 161 non-pregnant and 76 pregnant women with sufficient microbial reads, 143 and 71 remained. Further, for the sensitive analysis, we excluded samples from individuals who had any chronic somatic disease, currently smoked, or used illicit drugs, were on continuous medication, or had alcohol consumption, resulting in 73 and 42 in the non-pregnant and Pregnant groups, respectively.

The pregnancy study (PlaMi) was approved by the Independent Regional Research Ethical Committee at Karolinska Institutet, Stockholm (2015/2043–31/2) and complied with the Declaration of Helsinki^33^. All participants gave oral and written consent to participate. Everyone that chose to participate in the study received information about the study and signed a consent form prior to saliva collection. Saliva samples were obtained from 76 healthy pregnant women with full-term pregnancies (gestational age 37–42 weeks). Fifty were admitted for unlabored cesarean delivery (c-section) and 26 in active labor for vaginal delivery. The exclusion criteria were knowledge of fetal pathology, acute cesarean delivery and start of labor prior to cesarean. Data on health status and background were collected from the participants’ medical records. Other characteristics collected were age, BMI, smoking, parity, gestational age, international travel during pregnancy, antibiotic usage and delivery mode. The non-pregnant women participated in the MiMens study, sampling three saliva samples during menstruation, the follicular phase, and the luteal phase of their menstrual cycle. The study was approved by the Regional Committee on Health Research Ethics (H-17017580) and the Data Protection Agency in the Capital Region of Denmark (2012–58-0004) as detailed earlier ^1,3^. All 161 participants gave oral and written consent to participate. Briefly, the women were asked to come for a hospital visit on cycle day 1–3, 8–12 and 18–22. An ultrasound scan was performed to confirm that the participant was not pregnant. Exclusion criteria included pregnancy or an intention to become pregnant during the study, oligomenorrhea, irregular menstrual cycles, and antibiotics, anti-viral and anti-fungal medication in the previous two weeks before inclusion in the study or during the study period. Participants provided information about their lifestyle habits and medical history. Oral health and overall health status were assessed through questionnaires. Participants were asked about their oral health, including whether they had visited a dentist or dental hygienist in the past three months, received any plaque removal or caries treatment, or experienced any other oral health-related issues. We conducted a comparative cross-sectional study involving pregnant and non-pregnant women (Table S1, Table S2). To assess the robustness of our findings and minimize the impact of confounding variables, including smoking, snus or cannabis use, excessive alcohol consumption, recent antibiotic use, and chronic somatic diseases, the study design included a sensitivity analysis where these participants were excluded (Fig. 1) for secondary validation of the results. Furthermore, the study utilized qPCR to validate the abundance of selected oral bacteria identified via shotgun metagenomics.

## Saliva collection and processing

Saliva was collected following the established protocols^3,32^ . The women planned for cesarian delivery were asked to abstain from eating and oral hygiene practices overnight. Women who gave birth vaginally and non-pregnant participants were not allowed to eat, drink, smoke, chew gum or brush teeth 30 min prior to saliva collection. Whole saliva samples (2 ml) were collected by spitting into a SalivaGene Collector (Stratech Molecular GmbH, Germany) containing lyophilized DNA stabilization buffer, according to the instructions of the manufacturer.

## Extraction of salivary DNA and next-generation sequencing

Saliva aliquots of 600 µl were shipped to CoreBiome (OraSure, Bethlehem, PA, USA) where they were extracted with MO Bio PowerFecal (Qiagen, Hilden, Germany) automated for high throughput on QiaCube (Qiagen), with bead-beating in 0.1 mm glass bead plates. Three spaced negative controls and one positive control were included in each extraction. All negative extraction controls had undetectable amounts of DNA, and all positive controls were also approved. DNA concentration (for samples and controls) was quantified using Quant-iT Picogreen dsDNA Assay (Invitrogen, ThermoFisher Scientific, Carlsbad, CA, USA). Libraries were prepared using an adapted Nextera (Illumina Inc, San Diego, CA, USA) procedure and sequenced on an Illumina NextSeq using single-end 150 bp reads with a NextSeq 500/550 High Output v2 kit. Reads were processed with CoreBiome’s BoosterShot shallow shotgun sequencing technology. The raw sequencing reads are available from the European Nucleotide Archive under project PRJEB37731, samples SAMEA6662389-SAMEA6662857 (non-pregnant samples) and PRJEB38528, accession numbers ERX4191918-ERX4192266 (pregnant samples).

## Bioinformatic analyses

Human reads were removed by mapping to the hg19 release of the human genome using BBTools (available at https://sourceforge.net/projects/bbmap/). Because BoosterShot technology is optimized for fecal samples, taxonomy was re-annotated the reads using Kraken2 (26) with confidence set to 0.5 and Bracken, based on the Human Oral Microbiome Database v9.0.3. ^33,34^. Functional annotation based on modules was kept from the BoosterShot annotation^35^ Samples were excluded if they had fewer than 100 k non-human reads, were missing, or failed during collection. For non-pregnant subjects, all three samples per individual were required; if any sample was missing or failed, the entire subject was excluded from the analysis. As a result, four non-pregnant individuals were removed, while the remaining included subjects had all three samples retained. Taxonomic classification and relative abundances for selected samples are provided in Table S2. Functional module abundance profiles are summarized in Table S3.

## Real-time quantitative PCR (qPCR)

DNA samples were also analyzed using quantitative polymerase chain reaction (qPCR). The numbers four-selected target species of pregnancy associated bacteria were assessed in each sample using previously published primers^36,37^. Standard curves were generated using serial dilutions (10—0.00001 ng, 1:10) of bacterial DNA of known concentration (Table 1) and species-specific primers were used for *P. gingivalis*, *F. nucleatum, A. actinomycetemcomitans* and *P. intermedia*. Primers for *Streptococcus mutans* were custom designed (forward: 5'-TATGGTCTGCTGCCTGTTGC, reverse: 5'-TGCTACTGCCCATTACAATTCC). The qPCR experiments were performed in a total of 20 μL reaction volume, containing 8 μL of sample (diluted 1:4 in nuclease free water; standardized volume for samples = 2 μL), standards, or negative control (nuclease free water), 2 μL of primer solution (10 μM, 1:1 mixture of forward and reverse primer) and 10 μL of PowerUp™ SYBR™ Green Master Mix (Thermo Fisher Scientific). Samples were tested as single samples, standards and negative controls at least in duplicates. Amplification was performed using a 500/7500 Fast Real‐Time PCR System (Applied Biosystems) under standard PCR conditions (50 °C, 2 min), (95 °C 10 min), 40x (95 °C, 15 min), (60 °C, 1 min)) followed by melting curve acquisition. The obtained C_T_ values were then used to quantify the amount of bacterial DNA in each sample based on a linear standard curve (C_T_ vs log copy numbers) using the expected genome weight of each organism (representative reference strain, Genome Size NCBI). The detection limit for each experiment was defined by the mean C_T_ values of the negative control—1. Quality of the qPCR experiments was assessed for every bacterial species by testing three representative DNA samples in duplicates in each qPCR plate tested. Inter- and intra-operator reliability was calculated with variations of < 20% being considered acceptable. For the non-pregnant women, only samples from menstrual phase were tested by qPCR.

**Table 1:** Basic demographic characteristics of the participants.

	Non-pregnant	Pregnant	P value
Age mean (sd) [min, max]	24 (4.1), [18,40]	33 (5.7) [21,49]	< 0.0001
BMI*: mean (sd) [min, max]	22 (3.0) [17.6, 36.7]	24, (4.9) [18.5, 45.2]	0.01
Nulliparous n (%)	133 (93%)	37 (52.1%)	< 0.0001
Recent travel abroad § n (%)	84 (58.7%)	35 (49.2%) (30 missing)	0.003
Recent dental appointment § n (%)	42 (29.3%)	3 (4.2%) (36 missing)	0.02
Current smoker n (%)	31 (21.7%)	1 (1.4%)	0.0002
Current use of snus n (%)	14 (9.9%)	0	0.01
Current use of Cannabis n (%)	12 (8.5%)	0	0.03
Heavy drinker ¤ n (%)	46 (32.6%)	0	< 0.0001
Recent antibiotics § % n (%)	18 (12.1%)	9 (19.1%)^#^	0.4
Planned c-section n (%)	N/A	46 (64.7%)	-
Gestational age, weeks mean (sd) [min, max]	N/A	39 (1.2) [37,42]	-
Fasting	30 min: 143 (100%)	> = 12 h: 46 (64.7%) 30 min: 25 (35.2%)	< 0.0001

* for the pregnant cohort, BMI pre-pregnancy.

§ data not collected for vaginal deliveries

¤ more than 7 alcoholic units per week

% previous 3 months for the non-pregnant, anytime during pregnancy for the pregnant

^a^30 questionnaires missing, b36 questionanares missing

#24 missing

## Statistical analyses

All statistical analyses were conducted using R version 4.2.3. Alpha diversity within the oral microbiome was assessed using three primary metrics: Shannon’s Diversity Index, Pielou’s Evenness, and Observed Species Richness, using vegan (v2.6.4)^38^. Comparisons of diversity between pregnant individuals and non-pregnant individuals were made by evaluating the pregnant group against the average values across the three phases of the menstrual cycle in the non-pregnant group. Welch’s t-test was used for comparing continuous variables between groups, assuming unequal variances. All statistical tests were performed at a 95% confidence level, with a significance threshold of 0.05. Where applicable, multiple testing correction was applied using the Benjamini–Hochberg procedure to control the false discovery rate.

Beta diversity was calculated using Aitchison distances, computed after the addition of a pseudocount of 10^-6, using the vegdist function from Vegan (v2.6.4)^38^. These distances were evaluated using PERMANOVA (Permutational Multivariate Analysis of Variance) to test for significant differences between groups. The relative impact of confounding factors (Age, BMI, fasting period before sample collection, parity status, dentist visit, and travel abroad) on beta-diversity was also assessed and adjusted for where appropriate. Visualizations were generated using ggplot2 (v3.4.2) and vioplot (v0.4.0).

Associations between specific taxa or gene functions and the potential confounders were calculated in ANCOM-BC v2.0.1 (Analysis of Compositions of Microbiomes with Bias Correction), treating pregnancy status as a fixed effect and “participant ID” as a random effect^39^. We also conducted a multivariable analysis adjusting for fasting time, age, parity status, BMI, dentist visit, and international travel. Socransky’s color complex analysis was performed to assess differences in microbial community composition between pregnant and non-pregnant groups^40^. The relative abundances of microbes within the established Socransky complexes were compared, and group differences were assessed using a Mann–Whitney U test.

## Results

### Demographic data

From the original cohort of 161 non-pregnant and 76 pregnant women with sufficient microbial reads, 143 and 71 remained, respectively (Fig. 1). The pregnant cohort was older (mean 33 vs 24 years, p < 0.0001), had a higher BMI (mean 24 vs 22, p = 0.01), and was less likely to be nulliparous (52.1% vs. 93%, p < 0.0001). Additionally, the pregnant group had fewer recent dental appointments (4.2% vs. 29.3%, p = 0.02) and reported less recent travel abroad (49.2% vs. 58.7%, p = 0.003) **(**Table 1). The non-pregnant group had significantly higher prevalence of smoking, snus (moist tobacco) usage, alcohol and cannabis consumption, but not a significantly different prevalence in antibiotic usage (Table 1). Demographic characteristics of participants included in the sensitivity analysis are presented in Table S1.

### Microbial diversity and richness

**Fig. 2:**
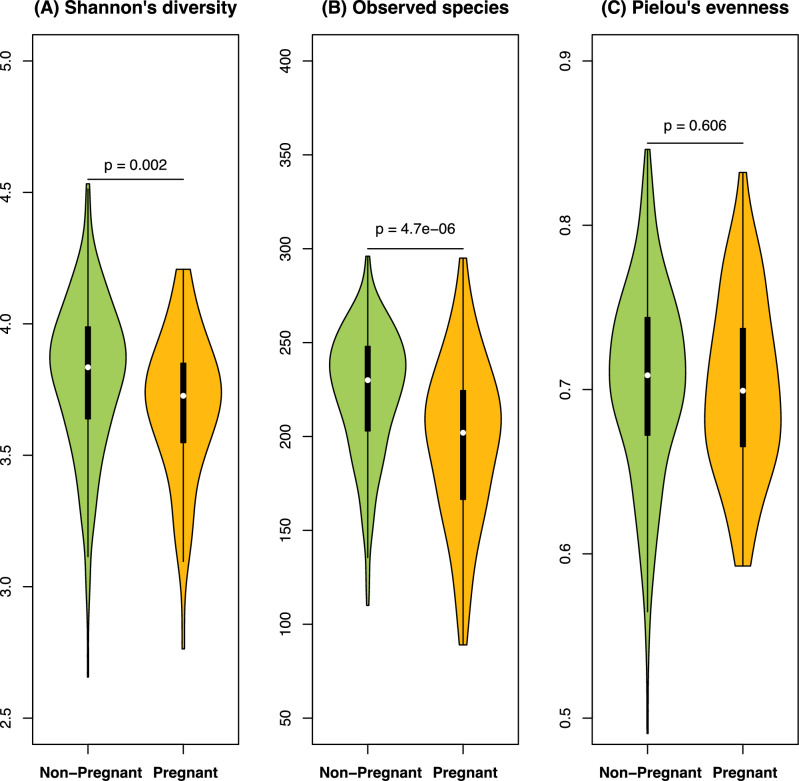
The pregnant women’s saliva had lower diversity and richness than the non-pregnant, but unaltered evenness. Violin plots representing the Shannon’s diversity (**A**), richness/observed species (**B**) and Pielou’s evenness (**C**) the salivary microbiota in pregnant (yellow color) and non-pregnant women (green color).

The saliva microbiome of the pregnant women had lower diversity than the non-pregnant women, which was driven by decreased richness (reflects number; fewer observed species) but unaltered evenness (reflects distribution) in the samples from pregnant women (Fig. 2). For the saliva samples from the pregnant women, the women planned for c-section (which had longer fasting time) had higher richness and somewhat lower evenness, than those undergoing a vaginal delivery, with an overall unchanged Shannon’s diversity (Fig. S1). These findings remained consistent even after excluding participants who reported tobacco, or cannabis use, recent antibiotic treatment, or other potential confounding factors (Fig. S2).

### Microbial composition

**Fig. 3:**
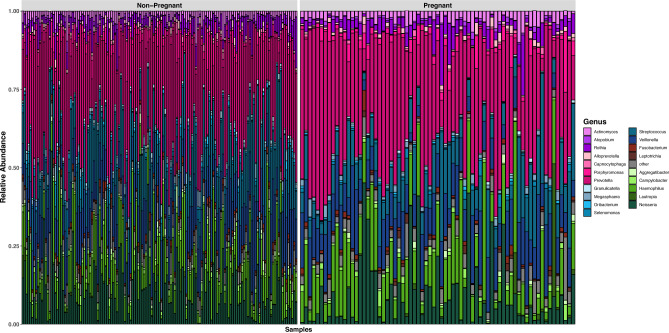
Taxonomic composition of the oral microbiota at genus level. Bar plot showing the relative abundance of the top 20 most abundant bacterial genera, with the remaining genera grouped as ‘Other’. Genera belonging to the same phylum share similar colour shades, with corresponding genus names indicated in the legend. Phylum-colour associations: Pseudomonadota: shades of green, Actinomycetota: shades of violet, Bacteroidota: shades of pink, Fusobacteriota: shades of brown, Bacillota: shades of blue, other phyla (Chloroflexota, Spirochaetota, Synergistota, Saccharimonadota, Mycoplasmatota): grey.

The bacterial distribution in the oral microbiome of pregnant and non-pregnant women was studied in terms of relative taxonomic abundance. There were 10 phyla, 102 genera, and 410 species identified. The phylum-level distribution of the oral microbiota for each pregnant and non-pregnant individual is shown in Fig. 3. The oral microbial species belonged to 10 phyla, namely Actinomycetota, Bacteroidota, Chloroflexota Bacillota, Fusobacteriota, Pseudomonadota, Spirochaetota, Synergistota Saccharimonadota and Mycoplasmatota*.* The samples separated on the second principal component (8.1% of variance) of PCA plot, mainly due to lower relative abundances of *Leptotrichia* spp.*, Neisseria* spp*.* and *Selenomonas* spp*.* among the pregnant women (Fig. S3). This difference was significant on PERMANOVA (r = 0.025, p = 0.001). The effect is partially attenuated but remains significant when adjusting for fasting time (r = 0.011, p = 0.001) and remains significant even after adjusting for multiple variables, including age, BMI, fasting status, dental visits, parity status, dentist visits, and international travel (r = 0.01, p = 0.001). These effects are also very similar when excluding participants with potential confounders (r = 0.026, 0.013, and 0.012, respectively; all p < 0.001).

### Abundances of selected oral bacteria in saliva of pregnant women

**Fig. 4:**
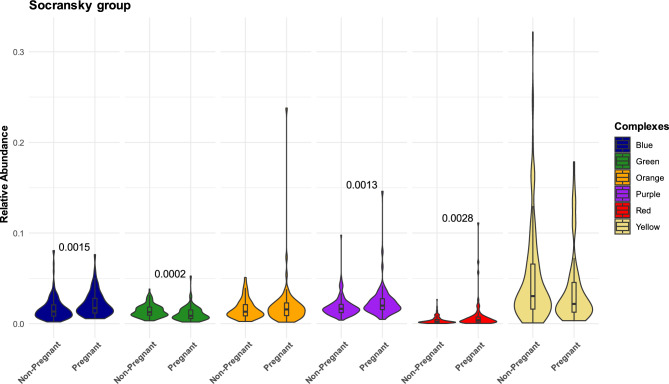
Analysis of oral microbial complexes in pregnant and non-pregnant women. Violin plot representing the the relative abundance of microbial complexes among the Socransky´s group between the pregnant and non-pregnant women samples. The Socransky complexes are six complexes of bacteria that commonly occur together, and color-coded as blue, green, yellow, purple, orange, and red. The blue, yellow, green, and purple complexes are compatible with oral health, whereas the orange and red complexes are correlated with inflammatory periodontal diseases. ^*^Significant difference are observed in red, green, purple and blue complexes. Red complex bacteria: *P. gingivalis*, *T. denticola*, and *T. forsythia*. The green complex bacteria: *E. corrodens*, *C. sputigena*, *C. gingivalis, and C. concisus*. The Purple complex bacteria: *V. parvula, A. odontolyticus.* The blue complex bacteria: *Actinomyces species* except A. *odontolyticus*.

Based on the color complexes of selected oral bacteria associated with different periodontal status^36^, we observed that the red, green, purple and blue color complexes are differentially abundant, with the red complex group showing higher levels among the pregnant women (Fig. 4), which is driven by *P. gingivalis* and *T. forsythia.* The differences in red and green complexes still hold when excluding participants with confounders (Fig S4). In contrast, pregnant women had lower overall abundance of health associated the green complex group **(**Fig. 4**)**, which seems to be mostly driven by *Campylobacter concisus* and, to a minor extent  *Capnocytophaga sputigena* (Fig S5B).

**Fig. 5:**
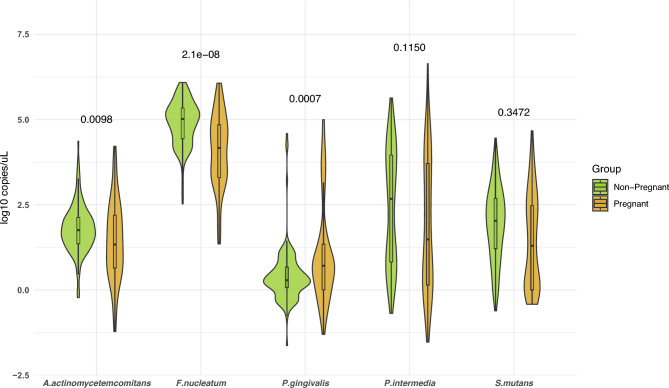
Abundance of selected oral bacteria in pregnant and non-pregnant women by quantitative real-time PCR Assays. Green: non-pregnant, Yellow: pregnant. Bacterial counts are reported in log_10_ scale. *A. actinomycetemcomitans*, *Aggregatibacter actinomycetemcomitans*; *F. nucleatum*, *Fusobacterium nucleatum*; *P. gingivalis, Porphyromonas gingivalis*; *P. intermedia*, *Prevotella intermedia*; *S. mutans*,* Streptococcus mutans.*

Moreover, we measured a few representative oral bacteria in saliva by qPCR. Quantitative analysis of *A. actinomycetemcomitans* revealed a significant difference in mean abundance between pregnant and non-pregnant women (p = 0.009; Fig. 5). In terms of relative abundance, *F. nucleatum* presented the largest difference being much lower among pregnant women (p = 2.1 × 10^–8^) (Fig. 5). *P. intermedia* and *S. mutans* were not significantly different, while *P. gingivalis* levels was increased compared to non-pregnant women (p = 0.0007) (Fig. 5).

### Differentially abundant taxa and predicted functions of salivary microbiota

**Fig. 6:**
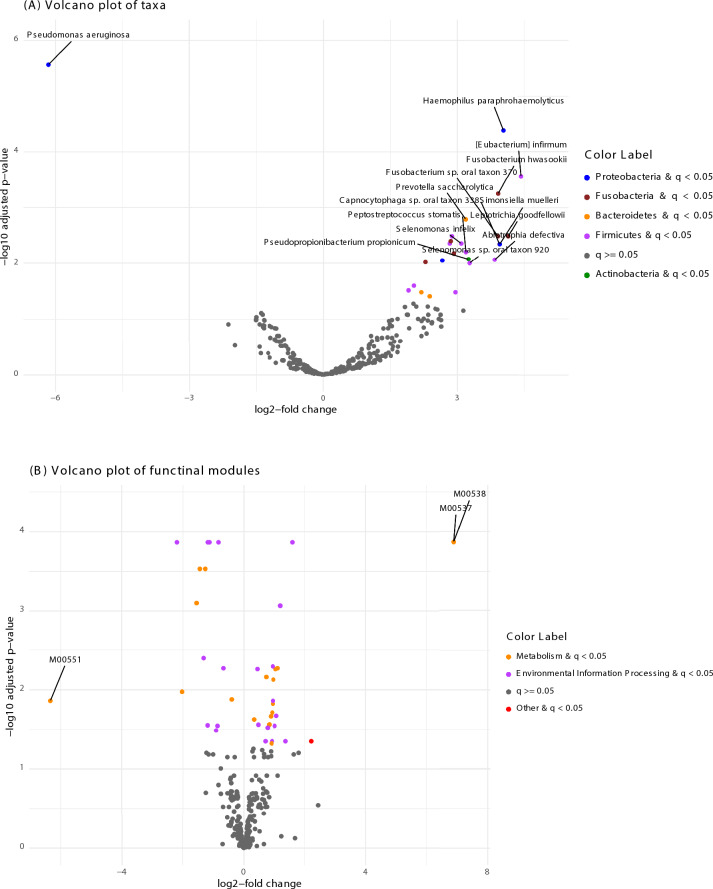
(**A**) Volcano plot of taxa differentially abundant between pregnant (> 0) and non-pregnant (< 0) women: The plot shows species fold changes on the X-axis and the negative logarithm (base 10) of the adjusted p value on the Y-axis. Grey dots represent species with an insignificant adjusted p value. Each coloured dot represents the species with significant adjusted p values and their corresponding phyla category, as shown on the left side of the figure. The labelled dots represent log fold change >  = 2 or = -2. (**B**) Functional modules differentially abundant between pregnant (> 0) and non-pregnant (< 0) women: Each coloured dot represents the modules with significant adjusted *p* values and their corresponding pathway category, as shown on the left side of the figure. The labelled dots represent log fold change >  = 2 or = -2.

In a hypothesis-free analysis of differential abundance, adjusted for age, fasting time, BMI, and parity, 25 taxa were found to be significantly different (q < 0.05) between groups after multiple testing correction. Of these, 13 taxa were more than three-fold higher in pregnant women, while only one taxon, *Pseudomonas aeruginosa*, was more than three-fold higher in non-pregnant women. (Fig. 6A; Table S4). Functional pathways for the bacterial communities in saliva were annotated against the ^35^database. 40 functional modules were differentially abundant (q < 0.05) according to pregnancy status (Fig. 6B; Table S5). Of these, the largest differences are M00551 (degradation of benzoate into catechol), which is six-fold higher in the non-pregnant group, while M00537 (degradation of xylene into methylbenzoate) and M00538 (degradation of toluene into benzoate) are six-fold higher in the pregnant group. These findings remained robust even after excluding participants who reported tobacco, snus, or cannabis use, recent antibiotic treatment, or other potential confounding factors. Table S5 includes the definition of each module.

## Discussion

The oral microbiome is a complex and dynamic microecosystem that fluctuates continually throughout the lifespan of a woman. Our results indicate that while the menstrual cycle affected salivary microbiome composition, the distinct patterns were driven by pregnancy. In concordance with prior studies, our findings reveal a reduction in microbial diversity and richness accompanied by a substantial shift in community composition in full-term pregnant women^41,42^. In addition to our taxonomic profiling, the functional analysis of these metagenomes indicated the emergence of a microbial functional core in pregnancy.

The reduced richness and diversity of the oral microbiota may indicate a compromised capacity of the microbial community to sustain homeostasis. Dysbiotic signatures have been increasingly tied to adverse pregnancy outcomes and oral bacteria like F. nucleatum and P. gingivalis have been linked to preterm birth, stillbirth, and placental inflammation via hematogenous spread and systemic immune activation24,43 Another study involving 40 women with mixed term pregnancies (25% preterm) found no shift in saliva microbiome throughout pregnancy^2^. The earlier work utilized a 16S rRNA gene-based analysis to characterize the pregnancy-associated oral microbiome^2,22^. The latter reported that an increase in microbiome diversity in the dental plaque samples could be linked to a ten-fold increase in progesterone and estradiol in pregnant women´s saliva^22^. Discrepancies between these findings and our results may reflect differences in the populations studied, the oral niches sampled, and methodological factors, including sample size. In the present study, comprehensive subgingival plaque sampling across the full dentition was not feasible. Instead, saliva was used as a surrogate matrix, which is widely accepted as reflecting the overall oral microbial community, as it contains microorganisms shed from multiple intraoral niches, including both supragingival and subgingival sites, as we and others have previously demonstrated44. Interestingly, while our findings suggest that confounding factors such as recent international travel, dental visits within the past three months, BMI, and age have limited impact on oral microbial composition during pregnancy, further studies are needed to link variations in the oral microbiome to specific factors such as oral hygiene and systemic health.

Imbalances in the normal bacterial equilibrium within the oral cavity, known as dysbiosis, can arise from an excessive proliferation of disease-associated bacteria or a reduction in health-associated bacteria29,45. P. gingivalis has been proposed as a keystone pathogen, in that it can exert a disproportionate ecological impact on the oral microbial community despite low abundance45. We have documented the proportional abundance of members of the Socransky red complex (*P. gingivalis, T. denticola and T. forsythia)* in the oral microbiome of pregnant women in line with previous observations, along with reduced levels of oral health associated *Capnocytophaga ie. C. sputigena*^22^. When the saliva samples were separated on the second principal component of PCA plot, this was mainly due to lower relative abundances of *Leptotrichia* spp*., Neisseria* spp*.* and *Selenomonas* spp*.* among the pregnant women which was consistent with the study by Balan et al^27^. Recent data also indicated that pregnant women who smoked demonstrated significant health-associated bacteria depletion in their oral microflora in the subgingival plaque including Neisseria, Veillonella and Actinomyces species15. In a previous case–control study, levels of oral bacterial species, including members of the red Socransky’s complex and A. actinomycetemcomitans, tended to elevate during pregnancy, particularly in mothers who experienced preterm birth43. Although A. actinomycetemcomitans levels were generally low (up to approximately 104 copies/µL), small quantitative differences were observed between pregnant and non-pregnant groups. These differences may, at least in part, reflect age-related variation between the cohorts, consistent with previous reports describing higher prevalence of A. actinomycetemcomitans in younger adults46.

Additionally, our findings revealed the presence of Fusobacterium and Prevotella species in the saliva of pregnant women, albeit at lower levels compared to non-pregnant individuals. This may not be surprising as previous studies showed that Prevotella levels were significantly higher during the luteal phase1,32. A more recent sequencing study by Paropkari et al. revealed elevated levels of Gram-negative facultative anaerobic bacterial species capable of estrogen metabolism in the subgingival plaque during pregnancy^15^. These alterations are hypothesized to be driven by pregnancy creating a nutrient environment that is more beneficial to those species. It is known that in the last phases of pregnancy, progesterone and estradiol levels increase to levels 10 to 30 times greater than during the menstrual cycle^47^. In vitro*,* estradiol and progesterone can both act as substitutes for vitamin K3 supporting the growth of *P. gingivalis*, and *P. intermedia*^48^. Moreover, estradiol can increase the formation of polysaccharides in oral biofilms as well as enhancing its coaggregation with other oral pathogens^10^. Notably, many bacteria have receptors for sex hormones, with some oral bacteria being capable of transforming or degrading these hormones^46^**.** Oral spirochaete *T. denticola* is capable of steroid metabolism and is known to possess 3β- and 17β-hydroxysteroid dehydrogenation activity^49^. Notably, *Prevotella dentalis* was significantly increased in pregnant women. Numerous species of *Prevotella* are typically found on different surfaces within the oral cavity such as and some *Prevotella* species could behave as true commensals but others as potential pathobionts^50^**.**

It is important to note that microbial shifts during pregnancy may not solely be attributed to the influence of sex hormones on bacteria, but also to the heightened oral inflammation experienced during this period^5,51^. Inflammation-driven alterations in microenvironments, such as those in the oral cavity, can impact the competitive dynamics and outcomes of microbial interactions, resulting in shifts in the relative proportions and diversity of species. Abundance of *P. denticola* and *P. gingivalis* have been associated with experimental gingivitis in pregnant women^20^. Similar results have been reported earlier that Socransky’s red complex bacteria were not present in pregnant women with healthy periodontium; however, it was present in pregnant women with gingivitis52,53. Reportedly, pregnant women who brushed their teeth less frequently tended to have higher levels of periodontal inflammation-associated bacteria in their oral cavity54. While the oral health status of the women in our study was evaluated solely through questionnaires, prior investigations have documented a higher prevalence of pregnancy gingivitis, ranging from 35 to 100%5,55,56 . Thus, elevated salivary levels of these species in pregnant women may, at least in part, reflect unmeasured gingival inflammation.

Another interesting finding was the higher abundance of *P. aeruginosa* in the saliva of the non-pregnant group. Although*,* it is a major respiratory pathogen and not commonly studied in oral cavity, its presence has been reported in saliva or dental plaque. This finding is in line with a previous study showing higher levels of *P. aeruginosa*^34,35^ have been found in young women at child-bearing age with gingival inflammation compared to those without evidence of gingivitis^56^. It is also possible that higher smoking prevalence among the non-pregnant women may contribute to differences in the bacterial composition of their saliva. Regular exposure to tobacco smoke has the potential to alter the oral microbiome in the oropharynx, potentially resulting in the chronic persistence of *Pseudomonas* strains^57^. Additionally, physiological and behavioral changes including alterations in salivary composition and flow, dietary modifications, and increased exposure to acidic conditions due to nausea may collectively contribute to an oral environment that favors the proliferation and metabolic activity of cariogenic bacteria such as S. mutans. In the present study, S. mutans levels did not differ significantly between pregnant and non-pregnant women. However, the low frequency of reported dental visits suggests that oral health status was not systematically evaluated clinically during pregnancy, and the presence of caries cannot be excluded in the absence of a comprehensive dental examination.

Furthermore, functional profiling demonstrated that specific functional modules differed significantly between the two groups, suggesting that pregnancy may influence both the structure and potential activity of the oral microbiota. Interestingly, our analysis at a functional scale revealed pregnancy-related changes in the number of reads for genes corresponding to "xenobiotic biodegradation and metabolism," which appears particularly intriguing. The potential for the degradation of xylene and toluene was increased in the pregnant group when compared to the non-pregnant group. Conversely, the abundance of genes related to benzoate degradation was greater in the non-pregnant group. These results were consistent in the sensitive analysis of the pregnant group; however, benzoate degradation was not observed in the non-pregnant group, suggesting it may be affected by other confounding variables. Previous studies have demonstrated that all four substances (snus, cigarette smoke, alcohol, and cannabis) perturb the oral microbiome and consistently enrich pathways for xenobiotic metabolism^58,59^. These exposures may therefore act as confounding variables influencing the observed differences in the non-pregnant group. Several species within the oral microbiome community have the metabolic capability to utilize hormones as sources of carbon and energy, processing them through degradation or chemical modification. In line with this, recent findings indicate that oral microbiome dysbiosis, particularly a reduction in health associated genera such as *Leptotrichia*, may contribute to pregnancy loss by impairing metabolic pathways (like L-arginine degradation) critical for vascular health and placental function^59^. Although our metagenomic analysis reveals the functional potential of salivary microbial community during pregnancy, it remains uncharted how this potential is translated to functional activity, as measured by the metatranscriptome or metabolome analysis.

Finally, this is the first large-scale shotgun investigation of the oral microbiota composition comparing pregnant and non-pregnant women with regular menstrual cycles in a healthy female Caucasian cohort. A strength of the cohort is the availability of a comprehensive questionnaire and journal data on the participating women that allowed for the selection of healthy individuals with a healthy lifestyle and compensated for the imbalance of several observed confounders. To address potential confounding, we adjusted for variables including age, BMI, fasting time, recent international travel, dental visits in the past three months, and parity. Sensitivity analyses further strengthened the study’s validity by excluding participants with known confounding factors such as smoking, excessive alcohol intake, snus or cannabis use, recent antibiotic use, and diagnosed medical conditions. These steps contribute to the robustness and reliability of our findings, minimizing bias due to residual confounding.

The main limitation of our analysis is its cross-sectional nature. The ideal study design would follow the same volunteers before, during and after pregnancy, which was not possible at this time. For this reason, it is not impossible that the results observed may be partially driven by the age of the volunteers or unknown individual characteristics. Another key limitation of this study is the homogeneity of the cohort, which consisted primarily of healthy Caucasian women. While this improves internal validity, it limits the generalizability of the findings, as we were unable to assess the impact of ethnic and socio-economic diversity on the oral microbiota. Another important limitation is the assessment of oral health. We evaluated dental health using self-reported questionnaire data; however, a large number of pregnant participants did not provide information on their recent dental visits. This lack of data restricts our ability to accurately assess oral health status, which could serve as a considerable confounding factor in microbiome studies.

In conclusion, these results confirm the existence of differences between the oral microbiome of pregnant and non-pregnant women with an overall lower diversity and richness of the salivary microbiome in women with full-term pregnancy. These results further suggest a connection between pregnancy and functional changes to the oral microbiome in women. As many of these changes are in a pro-inflammatory direction, further research is warranted to assess its potential impact on pregnant women and their newborns.

## Funding Declaration

The Centre for Translational Microbiome Research is partly funded by Ferring Pharmaceuticals (LE, IS-K). A research grant from Ferring Pharmaceuticals enabled the clinical recruitment and sampling (HN). The Rigshospitalet Research Fund (HN and MK), Karolinska Institute Strategic Funds (NB), the Swedish Research Council (2017–01,198, 2021–03,528 NB), and KI/SLL Strategic Dental Research Fund (NB, FoUI-966140). Data analysis was made possible by the SciLifeLab & Wallenberg Data Driven Life Science Program (KAW 2020.0239; LWH, ATA). The funders were not involved in the study design, collection, analysis, interpretation of data, the writing of this article or the decision to submit it for publication.

## Data availability:

Data from the pregnancy study is available at the European Nucleotide Archive (ENA) under project PRJEB38528, “Placenta biopsies, saliva samples, vaginal and rectal swabs collected at delivery for babies at term”, and samples and the non-pregnant participant’s samples are under project PRJEB37731, “Effects of the menstrual cycle and hormonal contraceptives on the human microbiome “, samples SAMEA6662389—SAMEA6662857.

## Supplementary Information

Supplementary Information 1.

Supplementary Information 2.

Supplementary Information 3.

Supplementary Information 4.

Supplementary Information 5.

Supplementary Information 6.
