# Supplementary material for: Shotgun metagenomic mapping of saliva reveals insights into diversity and function of the oral microbiome in pregnancy
Source: Sci Rep. 2026 May 27;16:16450. doi: 10.1038/s41598-026-54100-3 (PMC13216312; doi:10.1038/s41598-026-54100-3)
Supplement: Supplementary file 1 — Supplementary Information 1. [file 41598_2026_54100_MOESM1_ESM.docx]

**Supplementary Figures**

**Figure S1:** Violin plot representing A: Diversity (Shannon’s diversity), B: Richness (Observed species), C: Evenness (Pielou’s) in each delivery group and in different phases of menstrual cycle for the entire cohort. Orange: Menstrual. Brown: Follicular. Pink: Luteal. Blue: Vaginal delivery. Purple: C-section

**Figure S2:** Violin plot representing A: Diversity (Shannon’s diversity), B: Richness (Observed species), C: Evenness (Pielou’s) in each delivery group and in different phases of menstrual cycle, excluding smokers, heavy drinkers, cannabis and snus users and participants with known somatic disease.

Orange: Menstrual. Brown: Follicular. Pink: Luteal. Blue: Vaginal delivery. Purple: C-section

**Figure S3:** **Beta diversity analysis of salivary microbiota between pregnant and non-pregnant women.** The plot shows beta diversity calculated using Aitchison distance, visualized through Principal Component Analysis (PCA). PC1 and PC2 represent the first two principal components. Grey arrows with numbers indicate the species that most strongly influence the principal components. Each point represents an individual sample, with golden yellow circles representing non-pregnant women and purple triangles representing pregnant women. The numbered species and their corresponding names are displayed alongside the plot.

**Figure S4:** Violin plot representing the Socransky´s color complex relative abundances when excluding smokers, heavy drinkers, cannabis and snus users and participants with known somatic disease.

**Figure S5**: **(A) Violin plot representing the distribution of red complex microbes in pregnant and non-pregnant saliva samples.** Each colour shades represents specific species which is displayed on the right side of the plot. The relative abundances of *Tannerella forsythia* and *Porphyromonas gingivalis* were found to differ significantly between pregnant and non-pregnant women, with p values of 0.004 and 0.002, respectively.

**(B) The Socransky group's green complex microorganism distribution in saliva samples from pregnant and non-pregnant women.** Each shade of green represents a specific species, which is displayed on the right side of the plot. The relative abundance of *Capnocytophaga sputigena* and *Campylobacter concisus* were found to differ significantly between pregnant and non-pregnant women, with p values of 0.0001 and 0.001 respectively.

(C) **Violin plot representing the distribution of purple complex microbes in pregnant and non-pregnant saliva samples.** Each colour shades represents specific species which is displayed on the right side of the plot. The relative abundances of *Veillonella parvula* and *Actinomyces odontolyticus* were found to differ significantly between pregnant and non-pregnant women, with p values of 0.05 and 1.4e-^05^, respectively.
