# Supplementary material for: Shotgun metagenomic mapping of saliva reveals insights into diversity and function of the oral microbiome in pregnancy
Source: Sci Rep. 2026 May 27;16:16450. doi: 10.1038/s41598-026-54100-3 (PMC13216312; doi:10.1038/s41598-026-54100-3)
Supplement: Supplementary file 4 — Supplementary Information 4. [file 41598_2026_54100_MOESM4_ESM.docx]

|  | Non-pregnant | Pregnant | P value |
| --- | --- | --- | --- |
| Age  mean (sd)  [min, max] | 25 (4.8),  [18, 40] | 34 (5.7)  [21, 47] | **<0.0001** |
| BMI*:  mean (sd) [min, max] | 23 (2.7)  [18.5, 37.7] | 24, (4.3)  [17.9, 30.4] | 0.12 |
| Nulliparous  n (%) | 66 (90.4%) | 21 (50%) | **<0.0001** |
| Recent travel abroad §  n (%) | 45 (61.6%) | 18 (75%)  (19 missing) | 0.35 |
| Recent dental appointment §  n (%) | 20 (27.4%) | 3 (12.5%)  (18 missing) | 0.23 |
| Planned c-section  n (%) | N/A | 24 (57.1%) | - |
| Gestational age, weeks  mean (sd) [min, max] | N/A | 39 (1.3)  [37, 42] | - |
| Fasting | 30 minutes: 73 (100%) | >=12 hours: 24 (57.1%)  30 minutes: 18 (42.8%) | **<0.0001** |

**Table S1: Demographic characteristics of participants in the sensitivity analysis**

*** for the pregnant cohort, BMI pre-pregnancy**

**§ data not collected for vaginal deliveries**
